# Histone Posttranslational Modifications of CD4^+^ T Cell in Autoimmune Diseases

**DOI:** 10.3390/ijms17101547

**Published:** 2016-09-22

**Authors:** Zijun Wang, Heng Yin, Chak Sing Lau, Qianjin Lu

**Affiliations:** 1 Department of Dermatology, The Second Xiangya Hospital, Central South University, Changsha 410011, China; okvinci@126.com (Z.W.); kevinyin1984@aliyun.com (H.Y.); 2 Division of Rheumatology & Clinical Immunology, Department of Medicine, University of Hong Kong, Hong Kong, China; cslau@hku.hk

**Keywords:** posttranslational modification (PTM), histone modifications, T cell, autoimmune diseases

## Abstract

The complexity of immune system is tempered by precise regulation to maintain stabilization when exposed to various conditions. A subtle change in gene expression may be magnified when drastic changes are brought about in cellular development and function. Posttranslational modifications (PTMs) timely alter the functional activity of immune system, and work proceeded in these years has begun to throw light upon it. Posttranslational modifications of histone tails have been mentioned in a large scale of biological developments and disease progression, thereby making them a central field to investigate. Conventional assessments of these changes are centered on the transcription factors and cytokines in T cells regulated by variable histone codes to achieve chromatin remodeling, as well as involved in many human diseases, especially autoimmune diseases. We here put forward an essential review of core posttranslational modulations that regulate T cell function and differentiation in the immune system, with a special emphasis on histone modifications in different T helper cell subsets as well as in autoimmune diseases.

## 1. Introduction

**Figure 1:**
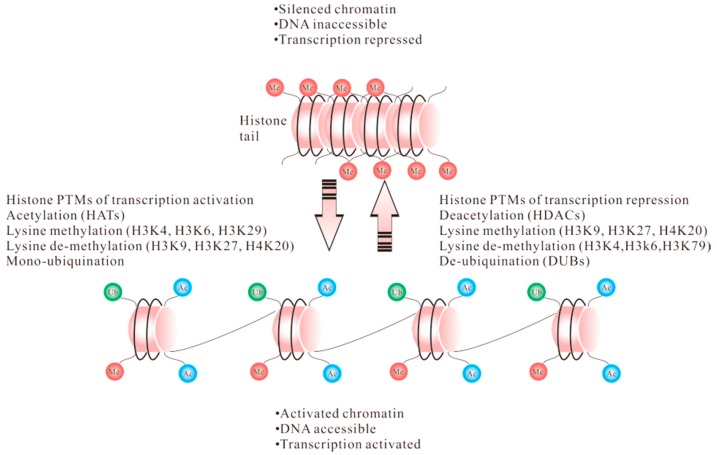
Histone posttranslational in transcriptionally activated and repressed chromatin.

The chromatin remodeling happens not only in the modifications themselves but also in the downstream events they produce for the protein-binding. This can be achieved when a histone code exists in the functional interaction among multiple histone modifications [1,2]. Although posttranslational modifications of chromatin remodeling are complicated, the significant donor is the modification of histone tails. A series of posttranslational modifications that are often generated on one or more histone tails, being a whole to form “histone codes” [3]. These histone proteins were once considered as dormant elements, but recent evidence have revealed that histones are integral and dynamic factors responsible for regulating gene transcription. The fundamental building block of chromatin structure is the highly conserved histone proteins, H2A, H2B, H3, and H4 [4,5,6]. Each of the core histones comprises a common domain known as histone fold, and it is this unit that permits histone–DNA interactions and for dimerization of histones [7]. A tetramer generated by the combination of the two H3 histones that conduct the interplay of the pairs of H3/H4 heterodimers. This tetramer is then linked to two H2B/H2A heterodimers with two four-helix bundles as well as histones H2B and H4, thereby giving the core octamer integrity [8]. These core histone proteins are often found enriched in lysine and arginine residues that can be altered to respond to external environments, thus permitting regulation of gene expression by affecting the intersection between DNA and the other chromatin elements. (Figure 1). 

The fundamental building block is presented as nucleosomal arrays consisting of nucleosomes with specific histones and combinations of different histone marks. Nucleosomes with various histone marks may differ from the several posttranslational modifications (such as acetylation, methylation, phosphorylation, and ubiquitylation). Certain posttranslational modifications (PTMs) of the histones can be linked with either activated or silenced chromatin. Transcription output, to some extent, is due to dynamic chromatin marks. Therefore, histone PTMs can be a determinant in the transcriptional process. Moreover, the adding or removing certain PTMs inside the gene may result in the totally different gene expression. A histone PTM that is generally considered to be activating can also be found in repressed genes. 

Histone is encoded by an elaborate collection of diverse posttranslational modifications including lysine acetylation, arginine and lysine methylation, phosphorylation, ubiquitination (Ub), proline isomerization, ADP ribosylation, arginine citrullination, sumoylation, carbonylation and biotinylation that directly or indirectly influence chromatin structure [9,10]. Among these modifications, acetylation and methylation are the most widely studied [2,11,12,13,14,15]. Functional features of these modifications have been implied in a wide range of cell development and differentiation. 

### 1.1. Acetylation

Histone acetylation refers to adding an acetyl group from one molecular element to histone, while the deacetylation is the opposite procedure in which an acetyl group is removed from a histone [16]. The process of transferring an acetyl group to lysine residues occurs in histone tail, and has the effect to eliminate the positive charge to the neutral on the histones. This action also has the potential to weaken the interaction of the nucleosome with the DNA backbone, which is negatively charged with phosphate groups. This may result in the transformation of a heterochromatin into the euchromatin structure, which is associated with the greater level of gene transcription [17,18]. 

Histone acetylation is typically regulated by two major kinds of enzymes, known as “histone acetyltransferase” (HAT) and “histone deacetylase” (HDAC) [19]. HAT molecules catalyze the transfer of an acetyl group while the deacetylation process is characterized by removing acetyl groups by enzyme known as Histone deacetylase (HDAC) [20,21]. Acetylation of terminal lysine residues is largely indicative of increased gene expression and usually associated with regions of actively transcribed chromatin. Conversely, deacetylation performed by HDAC molecules is associated with gene repression and is believed as a gene silencing process [21,22,23,24]. In this way, varieties of histone deacetylase inhibitors are now widely used as a means of increasing transcription in a hallmark of genes. 

Several lines of evidence in DNA sequencing technology have accelerated our acknowledgement of histone PTMs distribution patterns in the whole genome. To the best of our understanding, such advancements have suggested that among acetylated histones [25], histone H3 lysine 27 acetylation (H3K27ac) has been frequently delineated as being associated with active enhancer regulatory elements [26]. Specific histone PTMs, such as dimethylation and trimethylation at histone H3 lysines 9 and 27 (H3K9me2, H3K9me3, H3K27me2 and H3K27me3), are frequently established repressive chromatin states [27]. A tendency of increasingly rifeness of PTMs is discovered in histone protein as potential biomarkers, such as global difference in histone acetylation or H3K9 and H3K27 methylation, which are widely used in monitoring disease. 

### 1.2. Methylation

In contrast to acetylation, histone methylation is a complicated process, which is performed by two histone-modifying enzymes, histone methyltransferases (HMTs) and histone demethylases (HDMs). The methylation or demethylation processes are frequently found to occur at specific lysine or arginine residues on histones H3 and H4 [28]. Methylation activities that attenuate chemical interactions between histone tails and chromatin would accelerate the gene transcription process [29,30]. 

Methylation of histone tails may also play significant roles in gene activation or repression, and this lies dominant on what kinds of amino acids are modified, and the number of the methyl groups added to specific residues. It was shown that different transcriptional outcomes are determined by methylation of certain residues on histones that are differentially associated with constitution of the transcription machinery. For example, the addition of three methyl groups to histone 3 at lysine 9 (H3K9me3) or lysine 27 (H3K27me3) is tightly suggested to be linked to transcriptional repression, when it is upon regulatory control of certain histones at every residue. Nevertheless, dimethylation of histone H3 at lysine 9 (H3K9me2) is a signature for gene silencing. The occurrence of H3K4me3 and H3K27me3 on distinct genes is considered to be a specialization for poised expression, which means that the genes are not expressed at present but once activated by some specific signals, they will express immediately. 

### 1.3. Ubiquitination

Ubiquitination of histone is another process which is performed by adding ubiquitin peptides to lysine residues. In higher eukaryotes, the most abundant ubiquitin conjugations are ubiquitinated H2A and H2B. In chromatin, the ubiquitination of histones is frequently found at histone H2B and has been considered as participating in transcriptional elongation. This process are also known to contribute to the founding of other involved active signatures on the tails of histone H3 [31]. Ubiquitinated patterns of these histones are steady in vivo, and their interaction with nucleosomes is put forward to modify chromatin structure [32]. 

Even though the exact biologically functions of histone ubiquitination are remained largely obscure, identified information of this mechanism has implicated them many possible features which are strongly related to numerous cellular processes, such as DNA repairing, and cellular differentiation, and meiosis in higher eukaryotes [33,34,35]. Despite such an array of covalent modifications, ubiquitination has also been proposed as a highly dynamic modification which are determined by the activity of isopeptidases called de-ubiquitin enzyme that release the ubiquitin moiety and by the availability of free ubiquitin [36]. In spite of that distinct regulatory domains are not identified, mono-ubiquitylation has been noted as the most widely researched. The two most noteworthy modifications are H2AK119ub1, which is correlated with gene silencing [37], and H2BK123ub1, which is mentioned to be associated with transcriptional initiation and elongation [38,39].

### 1.4. Phosphorylation

Similar to the above modifications, histone phosphorylation has recently been discovered as another covalent modification of histones. The covalent modification of histones by phosphorylation has been suggested to be related to DNA repair, apoptotic signaling, and heat shock-induced pathways [40,41]. Histone phosphorylation takes place on serines, threonines and tyrosines and constitutes an essential part of the PTMs on chromatin [42]. Interestingly, phosphorylation of serine 10 of histone H3 has been indicated to be involved in altering chromatin structure and function, as well as regulating transcriptional activation, condensation of chromosomes during mitosis and meiosis, and regulation of cell division [41]. The degrees of phosphorylation are determined by kinases and phosphatases that add or remove the phosphate groups, respectively [43]. 

## 2. Posttranslational Modification of Histones in CD4^+^ T Cells

Histones are modulated by a series of posttranslational modifications, most of which are considered as a reader at each chromatin region and thereby link posttranslational modifications to cell destiny. In immune system, differentiation of CD4^+^ T cells is mediated by numerous modulatory control to specific cell related cytokine or nuclear transcription factor gene expression. This modulatory control is partly due to the regulation by histone modification which plays a central role in transcription action. In CD4^+^ T cells, it has been well established that conjugation between signaling pathways and environmental cues helps to maintain the cell identity. Such timely sensing of environmental signals promotes distinct differentiation processes and activates the required transcriptional landscape that is under control of histone PTMs. 

**Figure 2:**
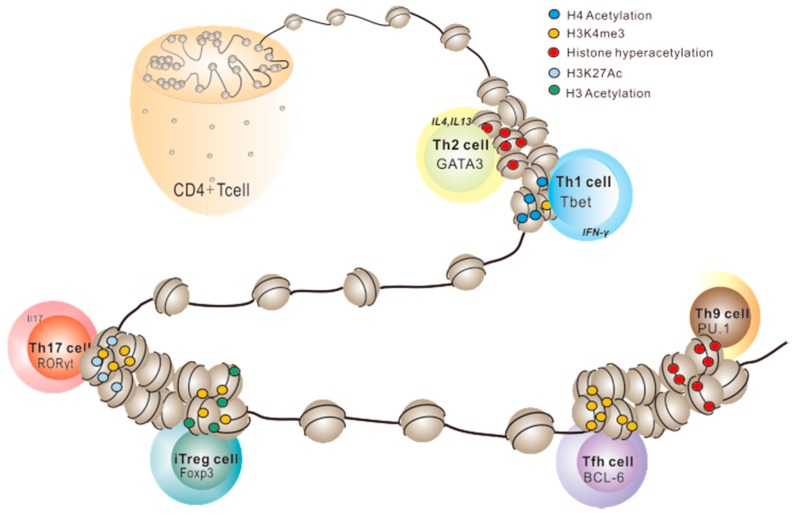
Dynamic histone posttranslational modifications in CD4^+^ T cells.

CD4^+^ helper T cells have been described as expressing cell-related core transcription factor (TF), which can be modified with a number of distinct marked cytokines [44,45]. It is widely accepted that the process when naïve CD4^+^ T cell differentiated in different subsets is dynamic. This is achieved by a network of strict regulation of transcriptional activity, thus enables them to achieve lineage integrity and stability. Following antigenic stimulation, the differentiation process of CD4^+^ T cells is performed after a combination with the antigen-MHC complex. Then they quickly proliferate and differentiate into distinct effector and memory subtypes depending mainly on antigenic stimulation. Moreover, differentiation towards different subsets of T helper cell is mentioned to play a role in regulating alternative cell fates. For example, fundamental function of a CD4^+^ T cell differentiation can be initiated when exposed to an infectious environment, the differentiated T cell lineages thus acquiring specific immune functions that are responsible for pathogen clearance. In naïve T cells, the specific gene locus exhibits a silenced (inactive or poised) pattern with some repressed modifications of key cytokines and TFs. (Figure 2). 

Histone modifications in Th1 and Th2 cell differentiation have been extensively analyzed at the Th1 (IFN-γ) and Th2 gene cytokine loci. In Th1 cells, Th1 cytokine gene (IFNg) gains permissive histone modifications (H3 and H4 acetylation, H3K4me3), whereas Th2 cytokine genes gain repressive histone modifications. Conversely, in Th2 cells, the Th2 cytokine locus gains active histone modifications while the IFNg locus gains repressive histone modifications. In Th17, iTregs, Th9 and Tfh cells, such histone marks are permissive and especially regulated in their active genes. H3/H4 acetylation and H3K4me3 are specifically increased in the IL-17, FOXP3, PU.1 and BCL-6 gene promoter, which permit the differentiation from Naïve T cell into different effector T cells.

### 2.1. Histone PTMs in Th1/Th2 Cell Development

The process when CD4^+^ T helper (Th) cells differentiated into Th1 cell subsets is featured by the production of Th1 specific cytokine interferon gamma (IFNγ), as well as the expression of the transcription factor T-bet. T-bet serves as an activator in regulating CD4^+^ T helper cell differentiation. However, molecular mechanisms that delineate stimulatory and inhibitory function of T-bet when regulating specific gene expression remain to be verified. Th2 cells are presented in part by expressing the major TF GATA-3 and producing Th2 specific cytokines, interleukin (IL)-4, IL-5, and IL-13. 

Th1 cytokine gene (IFNγ) and Th2 cytokine gene (IL-4, IL-5, and IL-13) loci are expressed in Th1 and Th2 cells, respectively, undergo structural and chromatin modifications during differentiation. T-bet and Gata3 are two primary transcriptional factors respectively responsible for the induction of Th1 and Th2 specific cytokines in the differentiation process towards Th1 and Th2 lineages. However, recently evidence has suggested that the production of such cytokines will not be altered when lack of the distinct transcription factors after the T helper lineages matured [46,47,48,49]. 

The Th1/Th2 cytokine gene loci have been researched extensively for gene regulation and lineage-commitment. Th1 and Th2 cells possess variable immune functions; Th1 cells are suggested to be associated with cell immunity resisted to intracellular bacteria as well as viral infection, while Th2 cells participate in humoral immunity and immunity against extracellular pathogens. Thus, dysfunction of Th1 and Th2 cell may initiate inflammatory cascade, which results in autoimmune disease and allergy, respectively. As is known to all, a number of multifunctional proteins are under regulation of PTMs and thus determine the cell fates by direct stimulation to target gene expression. 

The complicated process of Th1 and Th2 cell differentiation has been deciphered by histone PTMs and chromatin remodeling at specific loci [50]. Some differentiated Th1- and Th2-specific loci (IFNg and TBX21 for Th1 cells, and IL4, IL13 and GATA3 for Th2 cells) have been noted to obtain active histone marks and lose repressive marks. By chromatin immunoprecipitation, naïve CD4^+^ T cell always displays few histone acetylations at the promoter region of IFNγ locus as well as the IL4 and IL13 gene loci. However, the naïve T cells will obtain a large number of positive histone modification at the promoter of IFNγ locus or negative histone modifications at the promoter region of the IL4 and IL13 gene loci respectively, once upon an activation to differentiate into a Th1 subset. Positive histone modifications, such as acetylation of H4 (H4Ac) and H3K4me3 have been found on IFN-γ locus, while negative histone modifications, such as histone methylation have been found to silence Th2 cytokine (IL4 and IL13) gene loci. In turn, these modifications are completely exhibited in a different form when differentiated towards Th2 cells. IL4 and IL13 gene locus displays histone hyperacetylation while the IFN-γ locus presents negative histone modifications to silence the IFN-γ cytokine gene expression [51,52,53,54]. 

Histone acetylation is a positive histone modification which is associated with an active chromatin structure and is also suggested to be found at the promoters of IFN-γ locus and Th2 cytokine–IL4–IL13 locus in memory T helper 1 (Th1) and Th2 cells, respectively [55,56]. Several lines of evidence have reported the important role of Trithorax G, which can be activated by acetylation of histone H4 and this gene is a well-characterized chromatin modulator during Th2 cell differentiation [57,58]. STAT family is known as a modifier in the lineage-specific chromatin state. Induction of Trithorax G with STAT6 to displace PRC components in the Gata3 locus can drive Th2 cell development [57]. Moreover, STAT4 is essential to upregulate the level of permissive marks-H3K4me3 on specific genes of T helper 1 cell. While STAT6 lose the repressive marks-H3K27me3 in silenced genes of T helper 2 cells. Chromatin remodeling complexes have also been suggested as donors in maintaining the balance between permissive and repressive gene expression [59]. For example, H3K4-specific methylase MLL is essential in the maintenance of the expression and stability of IL4 and IL13 genes in Th2 cell [58]. 

It has been detected that a histone methylase SUV39H1 is involved in the trimethylation of H3K9 (H3K9me3), which can recruit heterochromatin protein to promote the stabilization of gene repression. This is interesting since the enzyme has been implied to silence Th1 gene loci expression and thus promoting the stabilization of Th2 cells [60]. Moreover, Ezh2 directly binds and facilitates appropriate expression of Tbx21 and Gata3 in the differentiation process of Th1 and Th2 cells, alongside with high levels of trimethylation at lysine 27 of histone 3 (H3K27me3) [61].In this study, a bridge has been built between Ezh2 histone PTMs and regulatory transcription of cell specific genes when ultimately differentiated into specific T cell lineage. 

### 2.2. Histone PTMs in Th17 Cell Development

T helper 17 (Th17) cell is characterized by expressing transcriptional factor retinoic orphan receptor γt (RORγt, encoded by RORC) as well as producing effector cytokines interleukin-17, IL-21, and IL-22. Th17 cells participate in host defensive systems against several harmful environmental factors, such as bacterial infection. The effector cytokines of Th17 cell act as bridges between the immune system and tissue damage thus playing pivotal role in tissue immunity. It has become increasingly appreciated that histone modifications, such as acetylation or methylation, are widely found at many Th17 cell-specific gene loci, including RORC, IL-23r, therefore playing significant roles in maintaining Th17 cell chromatin state [62]. The Rorc loci is indispensable for Th17 development and exhibits high levels of H3K27Ac and H3K4me3, which are two active regulations during Th17 cell differentiation [59], as well proved to be marked at the IL17a and IL17f loci [63]. 

There are many proteins that can identify histone modifications occurring in the chromatin, and therefore link the genetic material with transcriptional element. Such elements have been noticed in many studies. BET (bromodomain and extra terminal) protein is one of them, including a series of subtypes, BRD2, BRD3, BRD4 and BRDT. The BET protein is known to identify histones acetylation as well as involved in the differentiation process to Th17 lineage. Among this BET group, BRD2 and BRD4 are known to be attached to IL17a and IL17f locus precisely, thereby modulating diverse effector Th17 specific cytokines [64]. On the other hand, inhibiting BET proteins results in a disturbance in the differentiation towards Th17 lineage. In addition, the inhibition will suppress the activation of a fully differentiated Th17 cells. 

### 2.3. Histone PTMs in Treg Cell Development

Regulatory γT (Treg) cell is characterized by expressing main transcription factor Foxp3 and secreting cytokines IL-10 and TGF-β. It is well known that Treg cells are important to maintain immune homeostasis, and thus decreasing the regional immune response via a network of unclearly identified mechanisms. Foxp3 is requisite for Treg cell development and maintenance of Treg cell-mediated immunosuppression, and thus acquire immune homeostasis. Hence, chromatin regulatory mechanisms are suggested to regulate Foxp3 protein function. Posttranslational modifications (PTMs) of histones have been implicated to quickly change the gene expression level in many TFs, FOXP3 is among those TFs that can be modified with different kinds of histone posttranslational modifications, such as acetylation and methylation [65]. 

It has not been until recently that we have figured out that the differentiation process towards Treg cell is featured with histone PTMs in major gene activators, generated by FOXP3 [66]. Histone chromatin modifications develop a relatively permissive pattern in transcription activity, therefore increasing the combination of a transcription factor to lineage-specifying genes. In Tregs, permissive histone modifications such as H3K4me3 found at Foxp3 promoter region are indispensable for the Treg cell differentiation [67,68,69,70]. Another permissive regulation is histone acetylation displayed on the Foxp3 locus appears to be essential for Treg functionality and development. CBP and p300 are marked as histone acetyltransferases, and these two enzymes have been studied to maintain the development and function of Treg cells [71]. In Tregs, the absence of p300 is unable to survive and maintain the suppression function in autoimmune disease [72]. It is noteworthy that microbial metabolites such as short chain fatty acids maintain the homeostasis of colonic regulatory T-cell like HDAC inhibitors, resulting in enhanced exhibition of acetylation in the Foxp3 locus [73,74]. Notably, Treg-specific demethylation (TSDR) region is known for the recruitment of numerous permissive histone modifications. Conversely, the function of Foxp3 in iTreg is regulated by TGF-β signaling, in which H4 acetylation and NFAT are generated [75,76]. More recently, a study has found that the lack of a histone/protein deacetylases (HDACs), HDAC5 in CD4^+^ T cells can hardly differentiate into Treg cells under proper polarizing conditions. This strongly suggests that aiming at HDAC5 will limit the suppressive function and de-novo induction of Treg cells [77].

### 2.4. Histone PTMs in Other Effector T Cells

The description of excess CD4^+^ T cell subsets has enlarged our knowledge of Th cell differentiation beyond histone PTMs. Follicular helper CD4 T (Tfh) cells are specialized in germinal centers to help B cells to generate T cell–dependent B cell responses [78]. T follicular helper cells (Tfh) are known for helping B cell to produce antibodies and characterized by expressing the master regulator transcription factor Bcl6, as well as producing IL-21 cytokine [79,80]. Nonetheless, these two Tfh-related cytokine or TF are not stable as they are also be expressed by other immune cells, IL-21 is suggested to be secreted by Th17 cells, while Bcl-6 is central to the development of B cells. However, it is regrettable that few studies have deciphered the histone PTMs of Tfh differentiation. By ChIP-Seq analyses, Lu et al. has pointed out that Tbx21, Gata3 and Rorc loci display diverse active histone marks, such as H3K4me3, in Th cells which are similar to T follicular helper cell, but also ex vivo Tfh cells. [81].

Th9 cell is recently described as a new lineage of T-helper cells, which is defined by producing interleukin-9 (IL-9). Th9 cells might aggravate the immune response by increasing the level of antibodies. Additionally, it will also increase the number of infiltrated inflammatory cells in the respiratory tract. The high level of master transcriptional factor PU.1 (encoded by Spi1) has been linked to the Th9 lineage commitment [82]. 

A number of permissive or repressive histone modifications are noticed on the promoter region of PU.1 to switch the chromatin pattern from naïve CD4^+^ T cell to Th9 subset. Ramming et al. and his group have done a network of histone regulation patterns s in promoter regions of PU.1 in naive and differentiated CD4^+^ T cells. They have noticed a distinct and dynamic histone-modification alteration in promoter region at PU.1 gene which is associated with different state of T lymphocytes. Removal of negative histone mark, with a target repressor can increase the expression level of the Th9 major transcriptional factor PU.1. By contrast, inhibition of active histone acetylation, with the histone acetyltransferase inhibitor will in turn decrease expression level of PU.1 upon activation [82]. Another study has demonstrated that two transcriptional factors, Smad2 and Smad4, which are the products in the TGF-β signaling pathway, are necessary for Th9 differentiation. In the absence of Smad2 or Smad4, T cells may somehow carried out impaired IL-9, which is consistent with negative chromatin modification histone H3K27me3 on the IL9 locus [83]. The bridge between histone PTMs and PU.1 in the process of Th9 development needs further research. It is recognized that PU.1 is regularly deficient in the region where H3 K27 trimethylation –enriched. In addition, functionality of PU.1 is frequently mentioned as being related to permissive modification H3K4 dimethylation. 

## 3. Histone PTMs and Autoimmune Diseases

**Figure 3:**
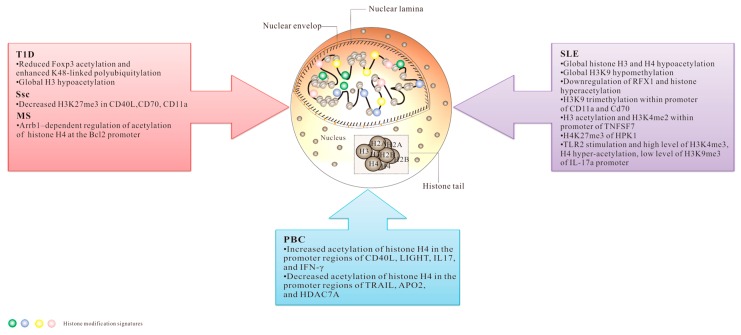
Histone posttranslational modifications involved in autoimmune diseases.

More recently, there is considerable evidence shows that histone PTMs are participated in various biological processes of the immune system, thus influencing the progression and pathogenesis of autoimmune diseases. Lack of the ability to maintain the stabilization of internal environment in autoimmune diseases is closely related to aberrant histone posttranslational modifications. Here we present an overview of the latest updates of histone PTMs in the regulation of some common autoimmune diseases, such as systemic lupus erythematosus (SLE), systemic sclerosis (SSc), multiple sclerosis (MS), primary biliary cirrhosis (PBC), and type 1 diabetes mellitus (T1D) (Figure 3).

This schematic representation illustrates the potential histone modifications, which can alter the cellular gene expression profile and thus promote diseases pathology. Chemical structures of selected compounds targeting histone modifications are also reported. A simplified scheme illustrating the structure of mammalian chromatin is also presented.

### 3.1. Systemic Lupus Erythematosus (SLE)

SLE is an autoimmune disease with multiple organs involved. It is characterized by production of autoantibodies targeted to a variety of nuclear antigens and deposit in organs and thus causing tissue damage by inducing inflammation. Although chronic autoimmune condition in SLE affects almost any organ system, the pathogenesis of SLE needs a further investigation due to the unclear mechanisms.

Posttranslational histone modifications are associated with the pathogenesis of SLE, as evidenced both in human patients and mouse models. A characteristic of genes are aberrantly combined with the presence of permissive and repressive histone PTMs in SLE. Even if histone acetylation effects on genes are not completely understood in SLE. CD4^+^ T cell profile of SLE patients with active disease are characterized by global H3 and H4 hypoacetylation in active lupus CD4^+^ T cells and disease activity correlates negatively with H3 acetylation as measured by SLEDAI. Furthermore, global histone H3K9 hypomethylation, but not H3K4, in both active and inactive lupus CD4^+^ T cells has been suggested [84]. Chromatin modifier genes under the regulation of histone PTMs have been observed to be aberrantly expressed in SLE. Such gene as SIRT1, CREBBP, P300, HDAC2, HDAC7, SUV39H2, and EZH2 have been documented in SLE CD4^+^ T cells [84]. Similar to CD4^+^ T cell of SLE patients, study on MRL/lpr mice has implied on the role of SIRTI as an important histone acetylation regulator, in which administration of SIRT1-siRNA into the MRL/lpr mice contributes to SIRT1 under-expression, which in turn resulted in the increased levels of global histone H3 and H4 acetylation in CD4^+^ T cells [85]. Moreover, differential expression of transcript level of histone methyltransferases (HMTs) and histone demethylases (HDMs) has been established in CD4^+^ T cells of MRL/lpr mice [86].

Several lines of evidence have performed many dysregulated transcriptional factors that are involved in SLE pathogenesis by recruiting histone PTMs. It has been shown that autoimmune responses in SLE may partly be explained by Regulatory factor X-box 1 (RFX1) downregulation, which results in histone H3 hyperacetylation at the promoter region of CD11a and CD70 genein CD4^+^ T cells of patients with systemic lupus erythematosus (SLE). Accordingly, downregulation of RFX1 contributes to the overexpression of CD11a and CD70, thus resulting an in increased autoreactivity in SLE [87]. Our further study has confirmed this function of RFX1. We have found that RFX1 can recruit a histone methyltransferase suppressor of variegation 3–9 (Drosophila) homolog 1 (SUV39H1) to the CD11a and CD70 genes promoter in CD4^+^ T cells. In this way, RFX1 can alter the level of histone modification H3K9me3 [88]. E4BP4 is another transcriptional factor studied in our group with altered histone modifications in SLE. By binding to the promoter region of CD40L, E4BP4 directly regulates the level of histone acetylation and methylation in the CD40L, thus influencing CD40L expression. 

In human, CD4^+^ T cells from patients with active SLE exhibit global histone PTMs. These PTMs are significant in regulating expression level of CD70 (TNFSF7) and CD40L gene, which are associated with autoreactivity of SLE. It is considered that aberrant histone modifications within the TNFSF7 promoter accompanied with other mechanisms lead the way to the development of lupus through elevating CD70 level in CD4^+^ T cells. Zhou et al. found that histone H3 acetylation and dimethylated H3 lysine 4 (H3K4me2) levels were significantly enhanced in TNFSF7 promoter in CD4^+^ T cells from patients with lupus, and these histone modifications correlated positively with disease activity. In addition, the MeCP2 protein levels in the TNFSF7 promoter were decreased in patients with active lupus [89]. By chromatin immunoprecipitation (ChIP) microarray data, Zhang et al. has identified significantly increased level of histone H3 lysine 27 trimethylation (H3K27me3) enrichment at the hematopoietic progenitor kinase 1 (HPK1), a negative regulator of T cell-mediated immune responses, promoter of SLE CD4^+^ T cells relative to controls. It seems that high levels of H3K27me3 at the HPK1 promoter may contribute to T cell overactivation and B cell overstimulation in SLE [90]. 

Despite the SLE related genes, receptors of the innate immune system also deciphered the function of histone PTMs in SLE T cells. Toll-like receptor 2 is tightly associated with histone modifications. An ex vivo experiment shows that upon activation, TLR2 in CD4^+^ T cells will elevate the level of both H3K4me3 and H4 acetylation in SLE patients. Additionally, the level of H3K9me3 is declined, in the IL-17A promoter, while the level of H4 acetylation is increased as well as a decreasing level ofH3K9me3in the IL-17F promoter region. It is more likely to believe that the overexpression of TLR2 accelerates immune reactivity and enhances expression level of IL-17A and IL-17F largely ascribed to the histone modifications in SLE [91]. Overexpressing of PP2Ac in mouse CD4^+^ T cells may partly results in SLE pathogenesis aimed at modulating IL-17 locus by increasing H3 acetylation via the activation of interferon regulatory factor 4. Therefore, dysregulation of PP2Ac has been known to be participated in the pathogenesis of SLE, mainly due to the promotion of inflammatory capacity of T cells [92]. 

### 3.2. Systemic Sclerosis (SSc)

SSc is an autoimmune fibrotic disorder featured with, microvascular dysfunction, and accumulation of the autoantibodies [85]. Although the exact etiology of the SSc still remains unclear, it is a multifactorial disorder that seems to be caused by complicated genetic mechanisms, environmental problems, which are crucial factors that may provide more explanations for pathogenesis of SSc. 

Recent studies have found that overall genome H3K27me3 level is decreased in the CD4^+^ T cells from SSc patients when compared with normal people. Specific genes like CD40L, CD70, and CD11a are indicated to be excessive activated in SSc patients due to the demethylation in their promoter, may also be regulated by the H3K27me3 pattern. This study also indicated an intriguing founding of JMJD3, which is one of the H3K27 demethylases, have been overexpressed in SSc CD4^+^ T cells. Therefore, there is a negative relationship between the mRNA levels of JMJD3 and H3K27me3 level. Taken together, decreased level of H3K27me3 contributes to the increased expression of JMJD3 in CD4^+^ T cells from SSc patients [93].

### 3.3. Multiple Sclerosis (MS)

MS is a human inflammatory demyelinating disease, characterized with autoimmune response against myelin proteins and progressive axonal loss by immune system reactivity that contributes to various stages of either relapsing or progressive neurological degeneration. 

The involvement of histone modifications in MS have been less well studied, although alterations in the major factors responsible for these modifications have been intensely evaluated. β-arrestin 1 (Arrb1) is a utility signaling factor which is essential to T cell survival. Studies have found an obvious increased expression of Arrb1 in CD4^+^ T cells from MS patients; Further, mice which lack the Arrb1 gene are presented to be more likely to survive from experimental autoimmune encephalomyelitis, which is a classical mouse model for multiple sclerosis. Accordingly, the enhanced expression of Arrb1 will be associated with the multiple sclerosis. It has been observed that overexpression of oncogene-Bcl2 is mainly due to the Arrb1–dependent modification of histone H4 acetylation at the Bcl2 promoter in an animal model of MS [94]. These results imply that histone acetylation level affect Bcl2 gene expression and is related to the pathogenesis of MS.

### 3.4. Primary Biliary Cirrhosis (PBC)

PBC is a chronic disease of liver, characterized by high titers of antimitochondrial antibodies and autoreactive T cells, which may cause destruction of the primary bile ducts, and ultimately developed into cirrhosis and liver failure [95]. Primary biliary cirrhosis is an autoimmune disease that primarily affects women.

As discussed in multiple sclerosis, overexpression of β-arrestin 1 in CD4^+^ T, as well as other T lymphocytes has also been documented in PBC. Studies have showed that elevated expression of β-arrestin 1 (Arrb1) occurred in T cells from PBC patients. Autoreactive T lymphocytes are frequently modulated by β-arrestin 1 (Arrb1), this may increase Arrb1 expression of β-arrestin 1, promote T cell proliferation; augment interferon generation, and decrease the functionalities of nuclear factor κB and AP-1. Plus, the regulation of Arrb1 in T cells is multifunctional and is also found to be involved in increasing histone H4 acetylation in CD40L, LIGHT, IL-17 and interferon-γ promoters, while decreasing histone H4 acetylation in the promoter regions of TRAIL, Apo2, and HDAC7A [96]. In this study, it has been demonstrated that Arrb1 can alter the gene expression of several disease related genes. Thus contributing to the pathogenesis of PBC, with variable mechanisms involved, including histone acetylation.

### 3.5. Type 1 Diabetes (T1D)

T1D is an autoimmune chronic disease, mediated by T cells. It is characterized by misrecognizing self-pancreatic cells for foreign agent and consequently damages the pancreas cells [97]. The two major types of diabetes are type 1 diabetes (T1D), and type 2 diabetes (T2D).

Several lines of studies have mentioned the critical role of histone modifications in the phathogenesis and development of T1D. In Treg cells of an autoimmune diabetes mouse model, the major transcription factor of Treg cell, Foxp3 has been suggested to lose the ability to respond to histone acetyltransferase-Tip60, and the histone deacetylase HDAC7. Additionally, FOXP3 from those impaired Treg cells of the mouse model is also regulated by other histone PTMs, such as Ikaros family zinc finger 4, Eos, which lead to decreased level of histone acetylation in Foxp3 promoter and increased level of K48-linked polyubiquitylation, these together, resulting in a decreasing number of Treg cells, thus influence the balance of the autoimmune system [98].

Latent autoimmune diabetes in adults (LADA) is a slow onset T1D. It has been identified that histone modifications displayed alternative gene expression to delineate how external environments s influence LADA. Liu et al. have shown a series of histone acetylation in CD4^+^ T cells from LADA patients. It has been observed a lower level of H3 acetylation in CD4^+^ T cells from LADA patients when compared with normal people .and this may help shed light on explaining the mechanisms with an epigenetic view in LADA. One interesting observation is that two LADA related clinically indications, the glycosylated hemoglobin (HbA1c) and GADA titer, are positively correlated with the H3 acetylation level in LADA CD4^+^ cells. Moreover, it has also been indicated that a decreased level of histone acetyltransferases CREBBP, as well as increased level of histone deacetylases HDAC1 and HDAC7, are observed in LADA patients. [99].

## 4. Conclusions

Histones make up the major part of protein constituent of chromatin, which are manipulated with many types of PTMs mostly occur on their tails. These modifications, taken together, comprise a “histone code” and could be applied to administrate genome or epigenome alterations, thus helping to establish a genetic or epigenetic crosstalk beyond DNA sequences. There are many histone PTM-associated molecules, which, accompanied with their related compounds, aim to evaluate specific functional states of chromatin, thereby regulating diverse chromatin-involved processes. A substantial number of recent studies demonstrate that these chromatin effector modules are associated with their unique histone PTMs. Pivotal characteristics in molecular interactions of histone PTMs have been summarized to shed a light on mechanisms performed in T cell biology and autoimmune diseases. Alterations of several histone modification patterns may have far-reaching meanings for biological processes, notably human diseases.
